# Investigation of the Implementation of Laser Surface Alloying of Cu with Cr–WC

**DOI:** 10.3390/ma15155396

**Published:** 2022-08-05

**Authors:** Justyna Domagała-Dubiel, Katarzyna Bilewska, Mirosława Pawlyta, Joanna Kulasa, Damian Janicki

**Affiliations:** 1 Łukasiewicz Research Network, Institute of Non-Ferrous Metals, Sowinskiego 5, 44-100 Gliwice, Poland; 2 Materials Research Laboratory, Silesian University of Technology, Konarskiego 18A, 44-100 Gliwice, Poland; 3 Department of Welding Engineering, Faculty of Mechanical Engineering, Silesian University of Technology, Konarskiego 18A, 44-100 Gliwice, Poland

**Keywords:** laser surface alloying (LSA), copper, Cr–WC powders, alloyed zone (AZ), microstructure, mechanical properties

## Abstract

This paper presents research on the microstructure and mechanical properties of an alloyed composite copper (Cu) surface layer, reinforced with a mixture of chromium–tungsten carbide (Cr–WC) powders. Copper alloying was performed using a high-power diode laser (HPDL). In the tests, three mixtures of powders with different percentage contents (75%Cr 25%WC, 50%Cr 50%WC, 25%Cr 75%WC) were injected into the melting pool during the laser surface alloying process. Microstructural evolution and the properties of the surface layer of copper after laser alloying were investigated. Structural investigations were performed using light microscopy, scanning and transmission electron microscopy (SEM, TEM) and X-ray diffraction (XRD). Microhardness and wear resistance of the modified surface layer were examined as well. After laser treatment the applied powders appear as uniformly distributed particles in the alloyed zone as well as nanoscale precipitates in the Cu matrix. Several types of precipitate characteristics, in terms of morphology, structure and chemical composition, were observed. Laser alloying of the surface layer modified the microstructure, which resulted in an increase in the hardness of the surface layers compared to the base material.

## 1. Introduction

Copper and its alloys are widely used as electric contact materials, in electronic devices, lead frame materials for integrated circuits, welding electrodes and overhead contact wires, mainly due to their high electrical and thermal conductivities. However, in many cases, the use of copper is limited by the unsatisfactory properties in the surface layer, mainly its low hardness. Low wear resistance affects the use and service life of components made of copper [1,2]. Therefore, some attractive methods are used in surface engineering to increase the performance of components and to reduce their wear. A significant improvement in anticorrosive properties and increase in resistance to abrasive wear can be obtained by applying metal surface layer shaping methods. Laser alloying, also known as laser surface alloying (LSA), is based on the introduction of alloying elements into the alloyed material [3]. The microstructure and properties of laser-alloyed surface layers depend on the selection of alloying elements and their composition. However, the LSA of Cu poses a great challenge due to its low laser energy absorption and high thermal conductivity. One way to improve the wear resistance of Cu is to incorporate a hard metal into the Cu matrix to increase the hardness while maintaining high electrical conductivity [4].

Laser surface treatment is an alternative to more conventional metal matrix composite (MMC) manufacturing methods. During laser surface alloying, the powder mixture together with the thin surface layer of the substrate is melted by a scanning laser beam. This leads to a rapid solidification and the formation of a surface layer. Rapid cooling and solidification of the laser-generated pool of molten material can result in the formation of various non-equilibrium phases and refinement of microstructure. If the powder injected into the molten pool contains particles that do not melt, an MMC surface is formed on the top of the substrate [5,6].

Laser surface alloying of copper and its alloys has been studied by several researchers. Manna et al. [7] attempted to alloy the Cu surface with aluminum (Al) (initially deposited by physical vapor deposition and then laser-alloyed) using a ruby laser. Dutta Majumdar and Manna [8] made an attempt to increase the wear and erosion resistance of Cu by means of laser surface alloying of Cr (electrolytically deposited with a thickness of 10 and 20 mm). Surface laser alloying increased the solubility of Cr in solid Cu up to 4–5 at % compared to the equilibrium solid solubility limit of 1 at %. It was found that the microhardness of the alloyed zone was significantly improved (up to 225 VHN) after laser surface alloying compared to 85 VHN of the substrate. Since hardness is related to Cr present in the solid solution and precipitated in the matrix, the change in the mean microhardness increases with the degree of solubility enhancement in the solid Cr in Cu.

In the study of [9], laser surface alloying of commercially pure copper (cp Cu) with chromium/titanium (Cr/Ti) and chromium/titanium/carbon nanotubes (Cr/Ti/CNT) was conducted using a high-power diode laser (HPDL). Compared to cp Cu (60 ± 10 HV_0.2_), the laser-alloyed specimens without and with CNT could achieve of hardness up to 423 ± 12 HV_0.2_ and 860 ± 24 HV_0.2_, respectively. Further, the wear resistance of laser-alloyed specimens was improved. The influence of laser alloying of titanium and silver powders on the microstructure and mechanical properties of copper was also investigated [10]. It was found that laser surface alloying with Ti powder increased the strength in the copper surface layer due to the formation of intermetallic phases (Cu_3_Ti_2_), while laser alloying with Ag powder changed the mechanical properties of the surface layer by solid solution strengthening.

Chromium in copper has influence with excellent anti-corrosion and mechanical properties (even at high temperature) without significantly reducing its thermal and electrical conductivity. The properties of CuCr alloys depend on the size of the chromium-rich phase, which may increase the hardness [1]. In general, a small fraction (0.7% wt.) of Cr dissolves in the Cu matrix to form a solid solution at eutectic temperature [11], where most of it is undissolved Cr in the Cu–Cr alloy with a high Cr content, which acts as the second phase.

Tungsten carbide combines advantageous properties, such as high melting point, good wettability by molten copper and, compared to tungsten, has a higher coefficient of thermal expansion (CTE) and lower thermal conductivity, making it a better choice as a reinforcing phase. Both carbon and tungsten have a very low solubility in liquid copper [11]. WC is widely used as a hard phase, which has been continuously and extensively researched for years [12]. WC–Cu composites are functional materials with excellent comprehensive properties [13,14,15].

In the present research, the Cr–WC surface layers with different Cr and WC contents were produced on copper substrates by laser alloying, and the effect of Cr and WC on the microstructure was investigated. Microhardness and wear resistance tests were also conducted.

The paper is organized as follows: Section 1 introduction to the topic. Section 2 contains a characterization of considered materials and experimental methods. Section 3 includes results with discussion, including: microstructure analysis by using light microscopy, scanning and transmission electron microscopy (SEM, TEM) and X-ray diffraction (XRD) and microhardness and wear resistance. Conclusions are given in Section 4.

## 2. Materials and Methods

**Figure 1:**
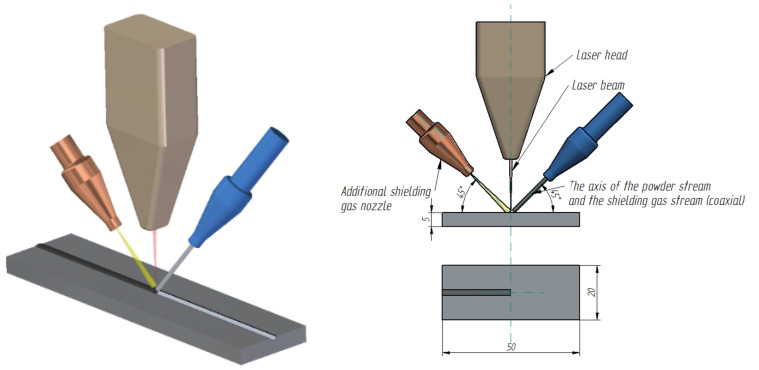
Scheme of laser machining setup (dimensions are given in milimetres).

**Figure 2:**
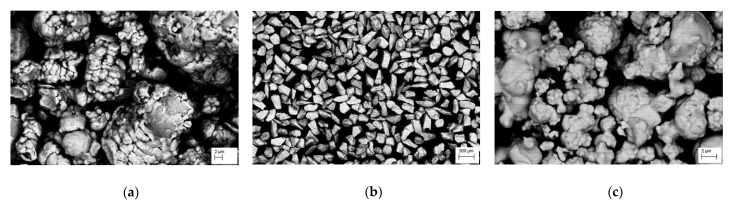
Morphology of powders: (**a**) Ni, (**b**) Cr, and (**c**) WC.

Commercially pure copper in the form of 50 mm × 10 mm and 5 mm high plates were used as substrates (Figure 1). Laser alloying of the surface layer of copper, using powder mixtures of Cr and WC with various weight percentage contents of 75%Cr 25%WC, 50%Cr 50%WC, 25%Cr 75%WC (Table 1) was carried out using a Rofin DL 020 high-power diode laser. Laser specifications are shown in Table 1. The morphology of the used powders is shown in Figure 2. The WC powder contained some amount of molybdenum (Mo) and nickel (Ni) (~1% wt.). As research has shown [15] Mo addition can strengthen the Cr phase.

Before the laser surface treatment, the samples were sandblasted with SiC particles to increase laser absorption. Then, in order to remove sandblasting particles from the surface, the samples were rinsed in an ultrasonic bath. The roughness of the sandblasted samples was Ra = 2.4 μm. Commercially pure Ni powder with average particle size below 10 µm was mixed with ethyl alcohol and then was preplaced in the form of a paste with a thickness of about 0.15 mm onto the sandblasted cp Cu substrate. The preplaced layer of Ni on cp Cu can prevent its high reflectivity [16,17,18,19].

In the second step, Cr–WC powders of different percentage concentrations were fed into the gas stream to the melt pool area. In order to protect the substrate against oxidation, the alloying process was carried out in protective gas.

In the experiments of laser copper alloying, continuous feeding of powder into the area of melt pool by dispensing the granulate by a feeder was applied. The powder feeder was connected to the transport gas cylinder and to the powder feed nozzle. Optimal parameters were selected on the preliminary test to ensure adequate melting of the injected powders and substrate with a minimum evaporation loss during the formation of the alloyed zone (AZ). The preliminary tests were performed for various laser parameters: laser power in a range of 1.0–2.0 kW, laser alloying speed: 0.1–0.15 m/min. Finally, laser surface alloying of copper was carried out at 2.0 kW laser power, 0.15 m/min laser alloying speed, and the powder feed rate was 2 g/min (Table 2).

The structure of the materials after laser alloying was observed with a light microscope and scanning electron microscope (SEM) equipped with an energy dispersive spectrometer (EDS). The microstructure of the samples was characterized using a S/TEM Titan 80–300 microscope (FEI Company, Hillsboro, OR, USA) equipped with an EDS spectrometer for chemical composition analysis. Crystal Maker (version 10.4.1) and Single Crystal software (CrystalMaker Software Limited, Oxfordshire, UK) were used to simulate the crystal structure and diffraction patterns. The structural data were obtained from Crystallography Open Database—Open-access collection of crystal structures of organic, inorganic, metal-organic compounds and minerals, excluding biopolymers [20]. Thin foils were made of about 1 mm thick samples cut with electrical discharge, which were mechanically thinned to a thickness of 0.2 mm, and then discs with a diameter of 3 mm were cut from them. The samples prepared in this way were subjected to mechanical thinning to a thickness of about 80 µm and then ionically polished.

The analysis of phase composition was carried out with XRD7 X-ray diffractometer from Seifert-FPM in Bragg–Brentano geometry. Co Kα characteristic radiation filtered by Fe foil was used. Patterns were collected in a range of 10–100° 2θ with 30 s per 0.04° 2θ step. Phases were identified in Seifert and Match! Software and 2019 PDF-4+ database from ICDD [21].

The microhardness of the formed surface layer after laser alloying was measured on a Vickers hardness tester with a 100 g load and 15 s counting time.

The abrasive wear resistance tests were carried out by means of a CSM Instruments high-temperature tribometer for wear tests at ambient temperature. The counter sample was in the form of a 100 Cr6 steel ball with a radius of Ø 6 mm, operating on a radius of 15 mm, at a speed of 50 cm/s and a load of 10 N, while the sliding distance was 1000 m.

## 3. Results and Discussion

### 3.1. Microstructure Analysis

In the present laser surface alloying process, high-power focused laser beam scans over the preplaced Ni layer with a Cr–WC powder mixture were simultaneously fed into the melt pool, melting the powders together with the substrate material layer. During melting (heating cycle) and solidification (cooling cycle) in the new surface layer, various phases in the laser-alloying layer are formed [22].

**Figure 3:**
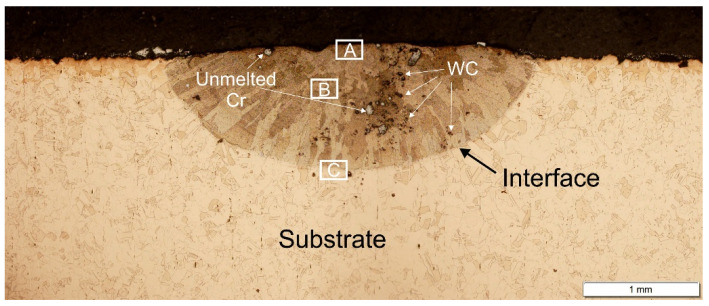
Macrostructure of the laser-alloyed surface layer Cr75WC25, single track.

Figure 3 shows an example image of a copper specimen with preplaced Ni powder after laser surface alloying with Cr–WC. The average LSA layer was about 900 µm. Single undissolved Cr powder particles are visible in the laser-alloyed layer. Absence of cracks and surface craters/ripples indicates that the process parameters represent the optimum LSA conditions of cp copper and a macroscopically homogeneous microstructure can be produced.

**Figure 4:**
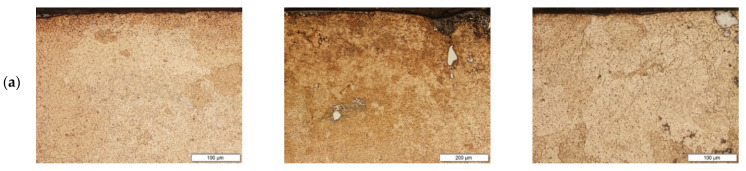

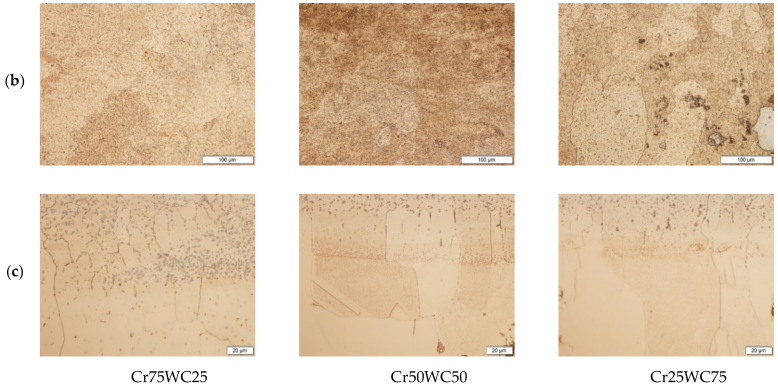
Cross-sectional images of LSA-treated samples with different percentage contents of Cr–WC, (**a**) upper area of the alloyed zone, (**b**) middle of the area zone, (**c**) boundary between surface layer and substrate.

Microstructure of the Cu after laser alloying with different percentage contents of Cr and WC is shown in Figure 4 in the upper area of the alloyed zone (area A in Figure 3), the middle (area B in Figure 3) and on the interface surface layer and substrate (area C in Figure 3). As seen in Figure 4, the microstructure consists mostly of fine grains with a few small precipitates—Cr-rich spheroids in the matrix and at the grain boundaries. During laser alloying on the specimens preplaced with Ni and feeding mixture, the Cr–WC powders melted instantly. The large temperature gradient (100–10,000 K/mm) between the center of the melt pool and the cooler solid liquid interface [23] and surface tension Marangoni fluid flow could lead to a mixing of the molten elements in the alloyed zone. There is a sharp transition at the interface between the surface layer and the substrate (Figure 4c). This microstructure is a result of a high solidification rate in the laser alloying technique. The Cu–Cr phase equilibrium diagram shows that solid solutions, of both Cr and Cu, have limited solubility in the solid state [24].

The formation of the Cr-rich spheroids in rapidly solidified Cu–Cr can occur through three different mechanisms, namely: liquid-phase separation (LPS) [25,26], primary growth of spherical grains [27] and precipitation from a supersaturated solid solution [28]. Owing to the existence of a liquid miscibility gap in the Cu−Cr binary phase diagram, the LPS could occur once a Cu−Cr liquid is undercooled into the gap. For that, a high cooling rate is required to allow the Cu−Cr liquid to drop into the miscibility gap and then separate into two new liquids. Each separated liquid Cr phase prefers to form a spherical shape to minimize individual surface energy.

It can be seen that a large number of spherical white Cr-rich phases is observed and well dispersed in the Cu-rich matrix. Laser rapid solidification can provoke a large dynamical undercooling and prevent the growth and coalescence of Cr-rich particulates [26].

The chromium content exceeding the eutectic composition in the laser-formed Cu samples is precipitated in the form of uniformly distributed spherical dispersive particles. The Cu matrix is in the form of a supersaturated solid solution, which is caused by rapid cooling. The surface layer of the alloying zone (AZ) of Cu consists of dispersed Cr-rich particles in the Cr-alloyed Cu-rich solid solution.

**Figure 5:**
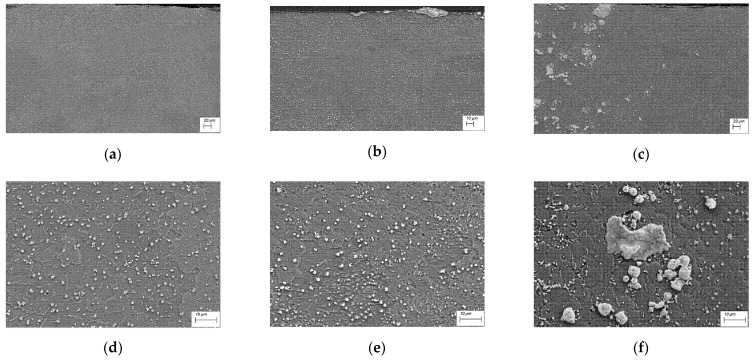
Copper surface layer after laser surface alloying with Cr–WC powder, laser power 2.0 kW, speed rate 0.15 m/min, (**a**,**d**) Cr75WC25, (**b**,**e**) Cr50WC50, (**c**,**f**) Cr25WC75.

Figure 5 shows a cross-sectional micrograph of the composite surface layer with different Cr–WC contents using scanning electron microscopy. Figure 5a–c display the microstructure of the upper part of the surface layer (Figure 5d–f enlarged areas from Figure 5a–c). In the melted zone, there are numerous small spherical particles rich in chromium (size approx. 200–400 nm). At the grain boundaries, these precipitates take a lamellar form. The surface layer of copper, after being alloyed with Cr75WC25 and Cr50WC50, shows areas with uniformly dispersed Cr particles (Figure 5d,e). The surface layer of Cu after alloying with Cr25WC75 laser powder is characterized by the least homogeneous microstructure. The undissolved WC particles can be separated in the laser-alloyed layer (Figure 5c,f). It can be observed that WC is unevenly distributed, concentrated mainly in the middle and upper parts of the surface layer. This is mainly due to the high melting point of WC and the large amount of heat absorbed, and the Marangoni convection effect is significant [29].

**Figure 6:**
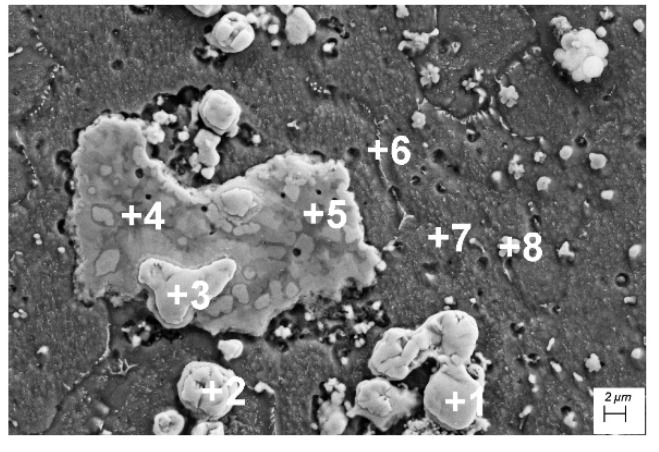
SE SEM image taken of the center area of the Cr75WC25 layer.

The SEM image presented in Figure 6 shows the microstructure of Cu after laser alloying with Cr25WC75. According to the EDS analyses of the points specified in Figure 6, the uniformly distributed fine precipitates are chromium and CrWMo-enriched phases (Figure 6 and Table 3). A composite structure is observed in the analyzed laser-alloyed surface layer with Cr25WC75 as agglomerates of WC particles in a copper-based matrix are obtained. They are visible as white spots against a dark background. These agglomerates display a very diverse structure and size. In the surface layer of the copper laser alloyed with a Cr25WC75 powder, there are areas with undissolved WC powder (points 1 and 2 of the analysis in Figure 6 and Table 3).

**Figure 7:**
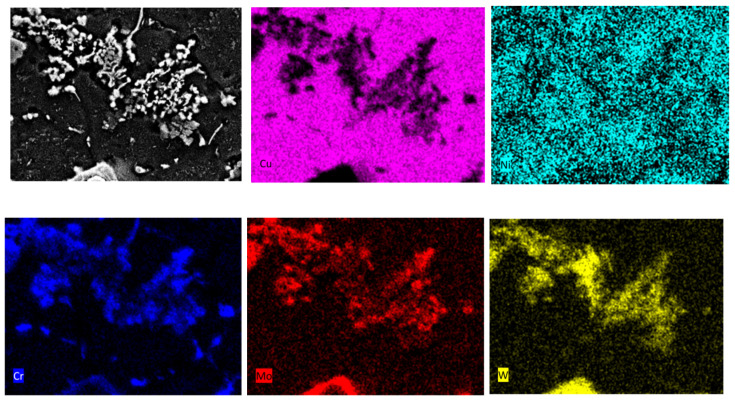
Surface microstructure and the corresponding EDS elemental mapping of Cu, Cr, Mo, W and Ni in alloying surface layer.

The microstructure of the copper surface layer after laser alloying with the Cr75WC25 powder and EDS elemental mapping of Cu, Cr, Mo, W and Ni are presented in Figure 7.

Regions rich in Cr, Mo and W are visible. The occurrence of this phase was confirmed by XRD analysis. These three elements form a solid solution, which corresponds to high hardness in this area.

**Figure 8:**
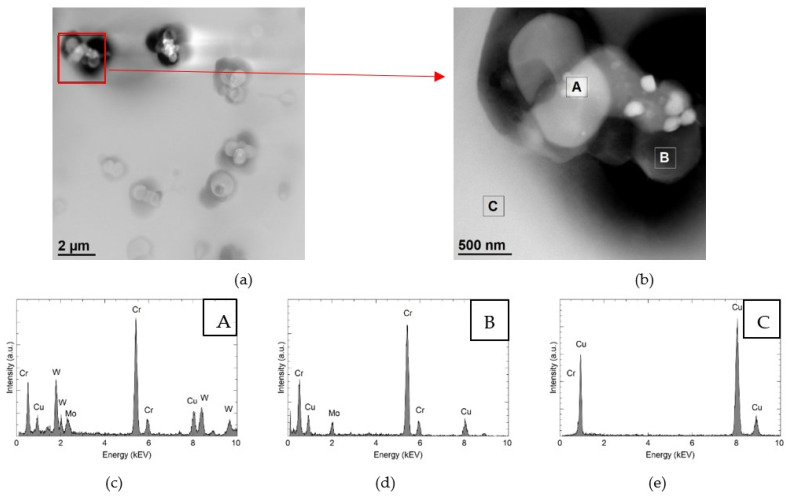
Structure of precipitates containing W and Cr in the sample Cr50WC50. HAADF images (**a**,**b**). Results of the chemical composition analysis in the areas marked A (**c**), B (**d**) and C (**e**).

The surface copper layer after laser alloying with Cr50WC50 powders shows precipitation in the form of aggregates of particles for different chemical composition. This is clearly visible in the High-Angle Annular Dark Field (HAADF) images obtained by transmission electron microscopy (TEM) (Figure 8a,b). HAADF images show chemical contrast (intensity is proportional to atomic number), so bright particles contain heavier elements. Precipitates containing W, Cr and Mo (Figure 8c) have a diameter of about 100–180 nm and are located on spherical particles containing Cr and Mo (Figure 8d). In the matrix, only the presence of Cu was confirmed by EDS (Figure 8e).

**Figure 9:**
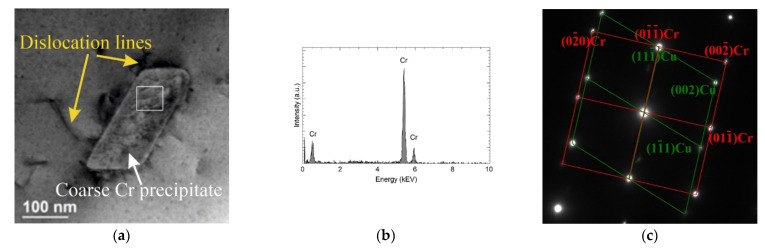
STEM-BF image of coarse Cr precipitate (**a**), the result of the chemical composition analysis (**b**), SAED from the [011] fcc Cu zone axis (**c**).

**Figure 10:**
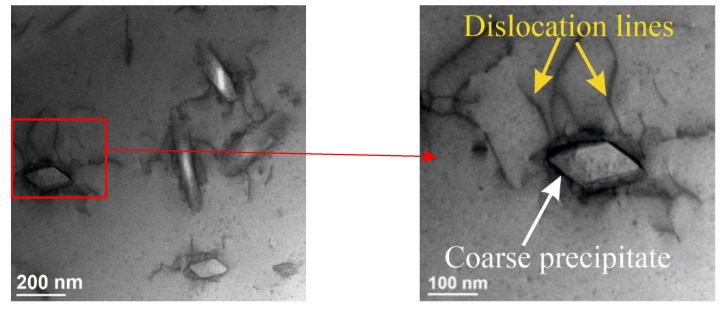
STEM-BF images of coarse Cr precipitates.

TEM was used to identify the phase composition of the observed precipitations. Several characteristic types of precipitates were observed—different in terms of morphology, structure and chemical composition. The first group is coarse chromium precipitates. They have a rhombic shape and a size of about 100 nm (Figure 9a and Figure 10). The chemical composition analysis confirms the presence of Cr (Figure 9b). Figure 9c shows the selected area electron diffraction (SAED) pattern obtained from the area visible in Figure 9a. The SAED was obtained in [011] Cu zone axis. The aperture (diameter 170 nm) selects both precipitates and the matrix fragment at the same time. Therefore, it was possible to identify the phase of the precipitate as well as the orientation with the matrix. The obtained results confirm the presence of the bcc Cr phase (COD ID: 9008531, space group Im-3m) and indicating the N–W orientation relationship with (face centered cubic) fcc Cu (COD ID: 9008468, space group Fm-3m) matrix [30]. The analyzed precipitates strengthen the alloy. STEM-BF images (Figure 10) show that they inhibit the movement of dislocations.

**Figure 11:**
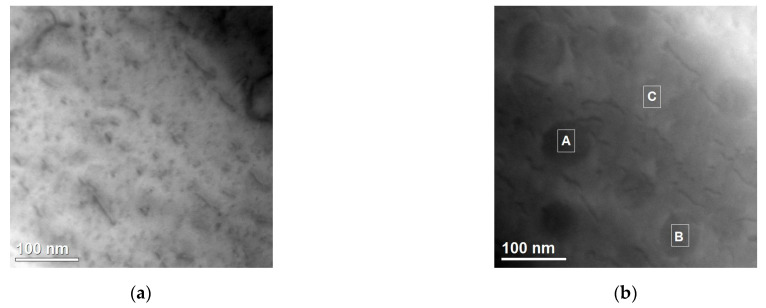

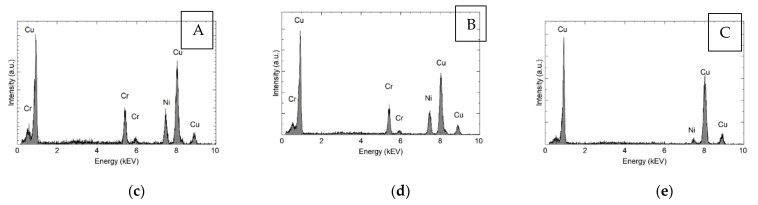
Structure of precipitates containing Cr and Ni in the Cr50WC50 sample. STEM-BF (**a**) and the HAADF image (**b**). Results of the chemical composition analysis in areas marked as A, B and C, respectively (**c**–**e**).

The second group consists of precipitates rich in chromium and nickel. They are round in shape. Their size is about 50 nm. These precipitates are not visible in the STEM-BF images (Figure 11) and they are poorly visible in the HAADF images. The lack of diffraction contrast indicates that they are coherent with the Cu matrix. The weak Z contrast confirms the difference in the chemical composition of the precipitates compared to the matrix material.

**Figure 12:**
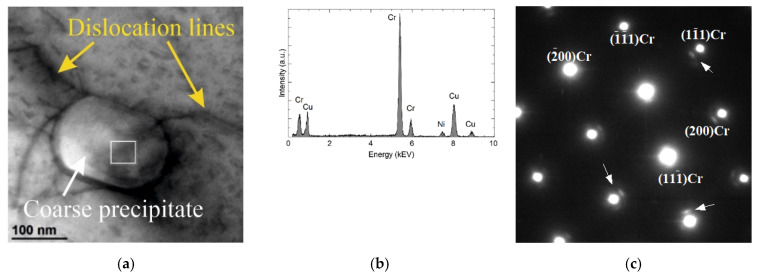
Structure of the precipitate containing Cr and small amount of Ni in the Cr50WC50 sample. The STEM-BF image (**a**) result of the chemical composition analysis (**b**), SAED of the fcc Cr in [011] zone axis (**c**).

Figure 12 shows an example of another type of precipitates. The precipitate is oval in shape and larger in size than previous ones. It also differs in chemical composition. The dominant component is Cr, but it also contains Ni and probably Cu. In this case, the diffraction pattern was identified as fcc Cr (COD ID: 9008467, space group Fm-3m) in [011] direction.

**Figure 13:**
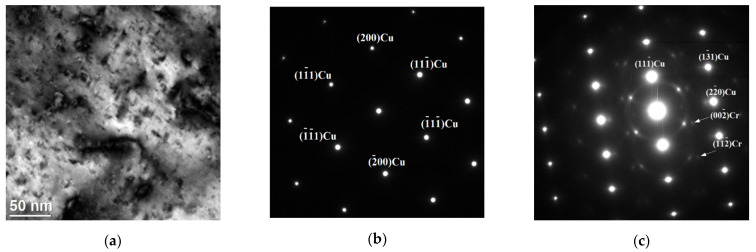
Bright-field TEM image of Cr50WC50 (**a**) SAED pattern from the [011] fcc Cu zone axis (**b**), SAED pattern from the [112] fcc Cu zone axis showing extra reflections from the ordered bcc Cr rich (the N–W relationship) (**c**).

**Figure 14:**
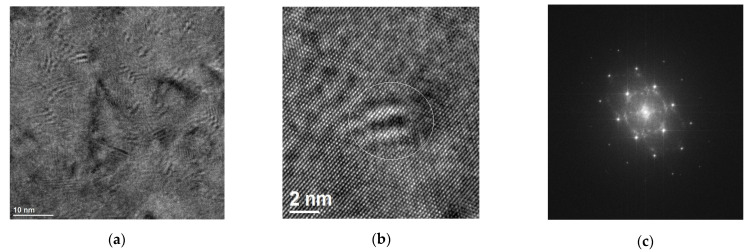
HR TEM image of Cr50WC50 obtained from the [011] fcc Cu zone axis (**a**), a magnified part from the previous image (**b**) and corresponding Fast Fourier Transform calculated form HRTEM (**c**).

Bright-field TEM image and corresponding SAED patterns of Cr50WC50 are shown in Figure 13. It indicates the presence of the ordered (body-centered cubic) bcc Cr-rich precipitates, which are in the N–W relationship with the matrix [31]. These precipitates are visible in HRTEM images (Figure 14).

These fine-dispersed particles in the matrix were necessary to obtain the desired strengthening effect. In addition, many dispersed nanoscale-precipitate phases existed in the Cu matrix and their morphologies included coffee-bean contrast and Moiré fringe contrast, as presented in Figure 14. According to previous studies, the precipitates with coffee-bean contrast are probably a Cr phase with fcc structure.

Cr precipitates that have nucleated during laser treatment are very small, typically only a few nanometers, and their OR with the fcc copper matrix usually induces strong local distortions. Similar results were obtained in [32], where early stages of chromium precipitations in copper alloy aged for 10 h at 713 K were analyzed at the atomic scale. In the analyzed sample, one can distinguish three kinds of contrasts associated with these precipitates. There were some typical coffee-bean contrasts, Moiré contrasts and larger fcc Cr precipitates. The coffee-bean contrasts are most probably fcc and coherent with the copper matrix, while others (Moiré fringes contrast) are probably bcc.

The unique properties of Cu–Cr alloys are mainly attributed to supersaturated solid solutions, metastable precipitates and Cr particles, etc., in the Cu substrate. It is noticeable that due to nanoparticles with lattice parameters similar to Cu, it hinders the unanimous agreement of several researchers on the evolution of the microstructure of the second phase particles. Fujii et al. [30] reported that Cr molecules can be classified into two types: flake-shaped and Moiré-shaped particles with bcc structure. Chen et al. investigated two types of phase transformations in Cu–Cr alloys. First, the continuous phase transition takes place in the aging stage below 300 °C. In other words, the dissolved atoms segregated on the surface of the crystal Cu matrix to form a GP zone with the fcc structure and then evolved into Cr particles with the bcc structure. Secondly, the dissolved atoms directly precipitated into Cr particles with the bcc structure at the aging stage above 300 °C [31,33]. L. Peng et al. [31] found that the precipitated sequence of Cu–Cr alloys is a supersaturated solid solution of Cu (Cr) → G.P zone with fcc structure (phase rich in Cr) → fcc Cr phase → ordered fcc Cr phase → bcc Cr phase.

**Figure 15:**
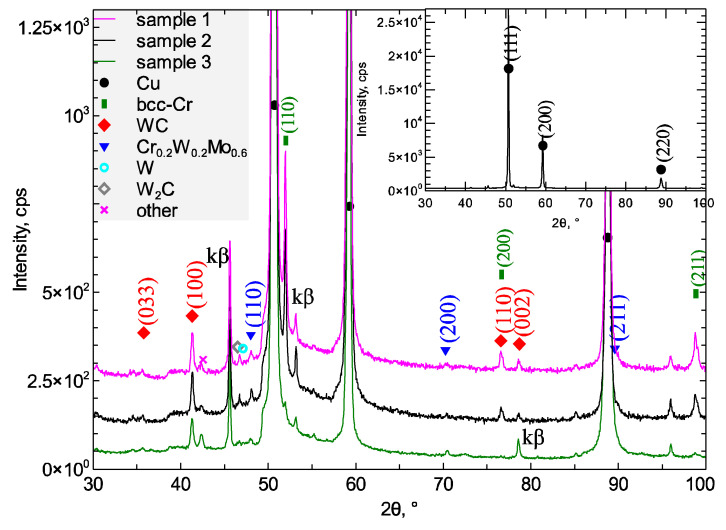
XRD pattern of Cu after laser alloying (sample 1: 75Cr25WC, sample 2: 50Cr50WC and sample 3: 25Cr75WC).

The examined samples have similar qualitative phase composition (Figure 15). The diffractograms of copper samples after laser alloying with 25Cr75WC, 50Cr50WC and 75Cr25WC powders show mainly two phases: metallic copper and metallic chromium. The highest intensity of chromium reflexes was observed for a copper specimen alloyed with Cr75WC25 powder and the smallest for a copper sample after alloying with the Cr25WC75 powder. In all samples, apart from the dominant copper phase, several other phases were identified. Most apparent is the cubic A2 structure of α-chromium. Due to low intensity and peak coincidence the structure of other phases is debatable. The most intense Bragg reflections of tungsten carbide W_2_C and metallic tungsten have similar positions in the 2θ scale. The main peak of the solid solution for the A2 lattice type corresponds to the composition of Cr_0.2_W_0.2_Mo_0.6_. In the WC phase, tungsten is supposedly partially substituted by other metals. Some other minor phases might include a mixed copper and chromium oxide. Nevertheless, atomic parameters were calculated for all phases considered. The upper layer of the copper substrate contains a solid solution of copper and, most probably, chromium at 0.9 % wt. in samples 1 and 2, and about 1.6 % wt. in sample 3 [34]. The lattice parameter in the α-Cr phase is slightly higher than for room-temperature pure metallic chromium structure (Table 4). It corresponds, for example, to 1–2 wt. % of Mo atoms dissolved in the chromium structure in samples 1 and 2, and about 1.5–2 wt. % in sample 3. The lattice parameters of metallic tungsten correspond to a pure W phase within the measurement error. The lattice parameters of molybdenum-based solid solution are slightly higher as compared to the data for phase of the Cr_0.2_W_0.2_Mo_0.6_ stoichiometry, which may indicate a higher content of larger atoms, such as molybdenum or tungsten. Cr-Mo solid solution is not identifiable due to peak overlapping with the copper substrate.

### 3.2. Microhardness and Wear Resistance

**Figure 16:**
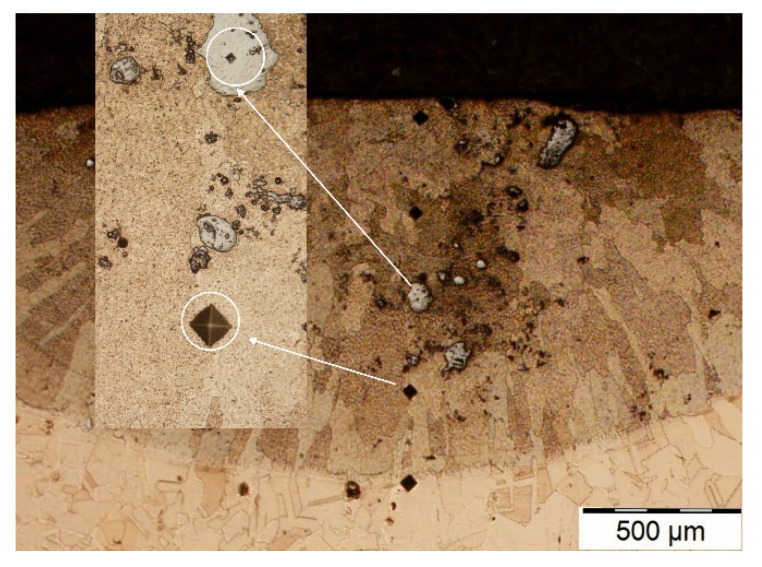
The cross-sectional microhardness of LSA Cr75WC25 samples.

The microhardness of the surface layer of copper after alloying with Cr–WC powder, with different powder concentrations, shows large scatter of its values at the cross-section of the melted zone. The large dispersion of the microhardness values on the cross-section of the remelted zone is caused by the presence of numerous fluctuations in the chemical composition in the laser-treated zone (Figure 16). The microhardness of the Cu matrix with spherical particles of Cr after laser alloying was in a range of 120–180 HV_0.1_. The microhardness of Cr particles is 200–400 HV_0.1_ while microhardness of the Cr-W and Cr-W-Mo precipitates (confirmed by EDS, XRD and HRTEM analysis) is within a range 700–1715 HV_0.1_. The microhardness of all laser-alloyed layers is much higher that of cp Cu (87.6 HV_0.1_). The Cr25WC75 surface layer of copper is characterized by the highest average microhardness, due to the greatest number of hard phases. The increase in hardness in the Cu surface layer after laser alloying with Cr is attributed to solution and dispersion hardening [8]. Manna et al. [35], as a result of laser alloying of copper with Cr powder, obtained a 2–3-times increased hardness; Hirose and Kobayashi [4] obtained a 1.5–2-times increased hardness.

Laser alloying of Cu samples with Cr–WC powder resulted in improved wear resistance. The tribological characteristics obtained in the pin-on-disk test of copper after laser treatment have similar wear track profiles. The reference samples were copper samples without laser treatment. The average coefficient of friction of copper samples alloyed with Cr–WC powder is in a range of 0.32 to 0.46.

**Figure 17:**
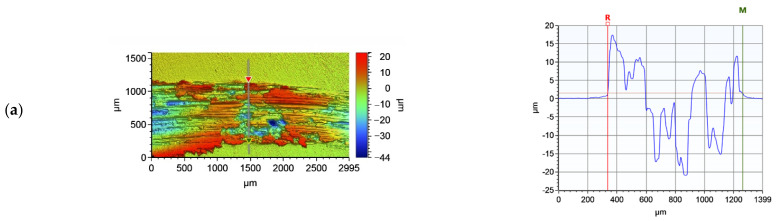

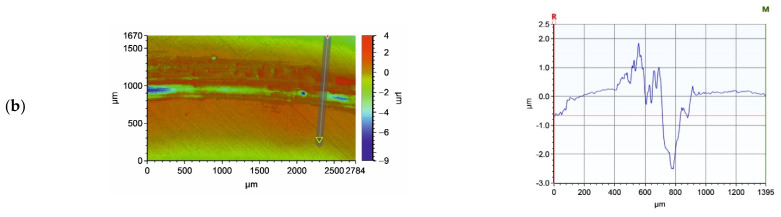
Representative 2D profiles of the wear track for (**a**) copper and (**b**) copper after laser alloying (Cr50WC50).

The wear track profile of copper and representative copper sample after laser treatment is shown in Figure 17. The wear depth of copper is more than twice as large as the wear depth of the copper surface layer after laser treatment.

The sample made of copper was characterized by a greater loss of mass, while in the case of the ball, the sample after abrasion of the copper surface layer after laser treatment was characterized by greater wear.

The wear track of the copper after the abrasive wear test is more than twice as wide as the wear track of the copper surface layer after laser treatment.

**Figure 18:**
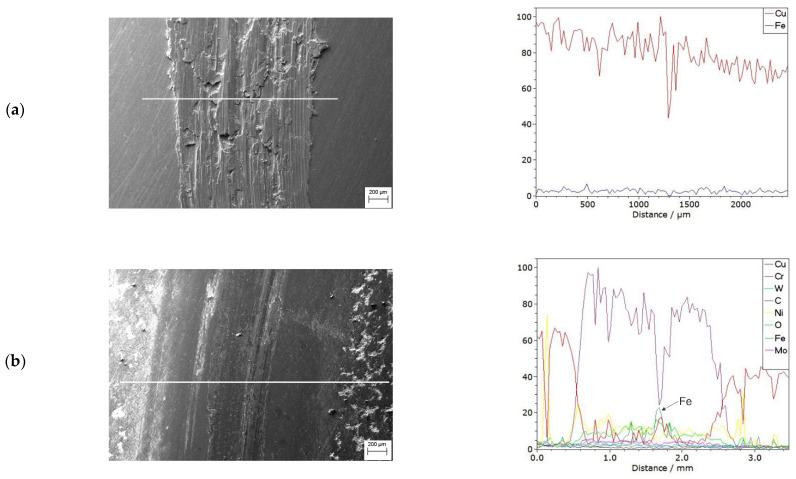
SEM images of the wear track and linear analysis of changes in chemical composition of (**a**) copper and (**b**) copper surface layer with Cr50WC50.

The linear analysis of changes in the chemical composition (Figure 18) showed that, for copper samples, only copper shows a trace of abrasion, while in the case of abrasion of the copper surface layer after laser treatment, the elements Cu, Cr, W, as well as iron come from the ball.

**Figure 19:**
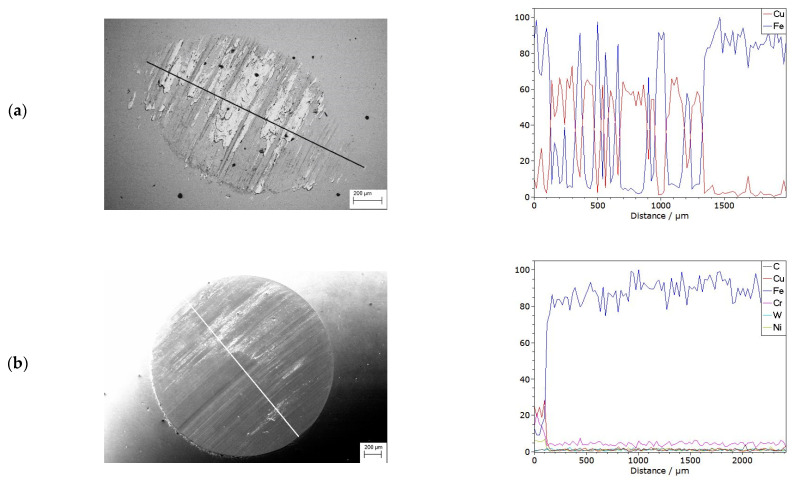
A trace of abrasion of a steel ball after a friction test and linear analysis of changes in chemical composition of (**a**) copper and (**b**) copper with laser alloying Cr–WC particles.

Linear analysis of changes in the chemical composition of copper, and copper after laser alloying with Cr50WC50, allows one to conclude that the ball was worn more in the friction process during testing on copper after laser surface treatment (Figure 19). A greater abrasion area in the sample was found during the wear of the copper surface layer after laser treatment.

During the testing of copper consumption, the mass is transferred from the copper sample to the counter sample, while in the case of the sample after laser treatment, the chemical composition of the counter sample did not contain any elements from the surface layer of the material subjected to wear.

The results indicate that enrichment of the surface layer with Cr–WC particles during laser treatment has a significant impact on the improvement in tribological properties.

## 4. Conclusions

Laser surface alloying of commercially pure copper with preplaced Ni powder, and simultaneously feeding Cr–WC powder, was successfully achieved using a high-power diode laser.

The conclusions are as follows:

1. The average thickness of the alloyed layer of copper with Cr–WC was about 900 µm.
2. Absence of cracks and surface craters/ripples in the alloyed layer of copper with Cr–WC indicates that the process parameters are: laser power—2.0 kW, alloying rate—0.15 m/min, and the powder feed rate—2 g/min, representing the optimum conditions for LSA of cp copper.
3. The copper top layer after laser treatment consists of the particles of used powders in the alloyed layer and nanoscale precipitates in the Cu matrix. Several characteristic types of precipitates were observed—different in terms of morphology, structure and chemical composition:
  - Precipitates containing W, Cr and Mo have a diameter of about 100–180 nm and are located on spherical particles containing Cr and Mo.
  - Coarse chromium (bcc Cr) precipitations with a rhombic shape and a size of about 100 nm.
  - Precipitates rich in Cr and Ni with round shape and a size of about 50 nm are coherent with the Cu matrix.
  - Oval precipitates—dominant components are Cr, Ni and probably Cu.
  - Fine-dispersed particles below 2 nm—precipitates with coffee-bean contrast are probably a Cr phase with fcc structure.
4. The surface layer of copper, after being alloyed with Cr75WC25 and Cr50WC50, shows areas with uniformly dispersed particles of applied powders. The surface layer of Cu after alloying with powder richest in WC (Cr25WC75) is characterized by the least homogeneous microstructure. The undissolved WC particles can be separated in the laser-alloyed layer.
5. The surface layer of investigated samples was found to be composed primarily of metallic copper. The crystalline phases detected in the samples’ surface layer after laser-alloying process include mainly bcc chromium, tungsten, tungsten carbide WC and a second bcc phase, corresponding to solid solution of Cr, W and Mo.
6. The surface layers of copper obtained as a result of laser alloying with Cr–WC powders are characterized by increased microhardness, which is 120–180 HV_0.1_ for Cu matrix with spherical particles of Cr and 200–400 HV_0.1_ for Cr particles while microhardness in the Cr-W-Mo precipitates is within a range 700–1715 HV_0.1_ compared to unalloyed pure copper (87 HV_0.1_). The large spread of the microhardness values in the laser-alloyed zone is caused by the presence of a number of chemical composition fluctuations. The increase in microhardness in the Cu surface layer after laser alloying with Cr–WC is attributed to solution and dispersion hardening. Due to the combination of non-equilibrium microstructures and the dispersion of hard phases, the surface properties are superior to that of copper not treated with a laser.

## Figures and Tables

**Table 1:** Technical specifications of HPDL Rofin DL 020 laser.

Parameters	Value
Wavelength, nm	808–940
Power range, W	100–2000
Focal length, mm	82
Power density range, kW/cm²	0.8–36.5
Laser beam spot dimensions, mm	1.8 × 6.8

**Table 2:** Composition of Cr–WC powders and laser parameters.

Specimen	Powder Composition		Laser Parameters	
	Cr (wt%)	WC (wt%)	Power, kW	Scanning Speed, m/min
Cr75WC25	75	25	2.0	0.15
Cr50WC50	50	50	2.0	0.15
Cr25WC75	25	75	2.0	0.15

**Table 3:** Chemical composition of the Cr75WC25 laser surface layers.

Elements	Composition in Weight, %							
	1	2	3	4	5	6	7	8
Cu	3.7	3.3		2.8	1.8	51.6	87.0	7.9
Cr	-	-	-	44.8	35.8	6.8	2.8	55.8
Ni	-	-	-	21.3	8.7	20.0	6.3	2.3
W	87.2	87.6	88.6	13.7	35.2	3.4	1.2	14.4
C	9.1	9.1	11.4	11.4	13.1	0.3	2.7	11.4
Mo	-	-	-	6.0	5.4	17.9		5.7
O	-	-	-	-	-	-	-	2.5

**Table 4:** Calculated lattice parameters of selected crystalline phases in comparison with the literature values.

	Lattice Parameters, a, Å, c, Å			
	75Cr25WC	50Cr50WC	25Cr75WC	Literature [21,34]
Cu (γ1 phase) (cubic Fm-3m #225)	a = 3.6173 ± 0.00005	a = 3.6174 ± 0.0001	a = 3.6194 ± 0.0002	a = 3.6146 (20 °C)
Cr (α phase) (cubic Im-3m #229)	a = 2.8868 ± 0.0007	a = 2.886 ± 0.001	a = 2.888 ± 0.005	a = 2.8846 (20 °C)
(W)C qusongite (hexagonal, P-6m2 #187)	a = 2.925 ± 0.002 c = 2.9475 ± 0.05	a = 2.9244 ± 0.003 c = 3.0258 ± 0.3	a = 2.9293 ± 0.002 c = 2.9781 ± 0.007	a = 2.90 c = 2.93 (25 °C)
W (cubic Im-3m #229)	a = 3.188 ± 0.01	a = 3.185 ± 0.016	a = 3.207 ± 0.013	a = 3.1650 (25 °C)
Cr_0.2_W_0.2_Mo_0.6_ (cubic Im-3m #229)	a = 3.1159 ± 0.007	a = 3.111 ± 0.011	a = 3.1158 ± 0.002	a = 3.11 (25 °C)

## Data Availability

Data sharing is not applicable to this article.
